# Need for a Paradigm Shift in the Treatment of Ischemic Stroke: The Blood-Brain Barrier

**DOI:** 10.3390/ijms23169486

**Published:** 2022-08-22

**Authors:** Maria Luz Alonso-Alonso, Ana Sampedro-Viana, Sabela Fernández-Rodicio, Marcos Bazarra-Barreiros, Alberto Ouro, Tomás Sobrino, Francisco Campos, José Castillo, Pablo Hervella, Ramón Iglesias-Rey

**Affiliations:** 1 Neuroimaging and Biotechnology Laboratory (NOBEL), Clinical Neurosciences Research Laboratory (LINC), Health Research Institute of Santiago de Compostela (IDIS), 15706 Santiago de Compostela, A Coruña, Spain; 2 NeuroAging Laboratory Group (NEURAL), Clinical Neurosciences Research Laboratory (LINC), Health Research Institute of Santiago de Compostela (IDIS), 15706 Santiago de Compostela, A Coruña, Spain; 3 Translational Stroke Laboratory (TREAT), Clinical Neurosciences Research Laboratory (LINC), Health Research Institute of Santiago de Compostela (IDIS), 15706 Santiago de Compostela, A Coruña, Spain

**Keywords:** blood-brain barrier, ischemic stroke, nanoparticles, neuroprotection, stroke prevention

## Abstract

Blood-brain barrier (BBB) integrity is essential to maintaining brain health. Aging-related alterations could lead to chronic progressive leakiness of the BBB, which is directly correlated with cerebrovascular diseases. Indeed, the BBB breakdown during acute ischemic stroke is critical. It remains unclear, however, whether BBB dysfunction is one of the first events that leads to brain disease or a down-stream consequence. This review will focus on the BBB dysfunction associated with cerebrovascular disease. An added difficulty is its association with the deleterious or reparative effect, which depends on the stroke phase. We will first outline the BBB structure and function. Then, we will focus on the spatiotemporal chronic, slow, and progressive BBB alteration related to ischemic stroke. Finally, we will propose a new perspective on preventive therapeutic strategies associated with brain aging based on targeting specific components of the BBB. Understanding BBB age-evolutions will be beneficial for new drug development and the identification of the best performance window times. This could have a direct impact on clinical translation and personalised medicine.

## 1. Introduction

Nowadays, neurological diseases are considered the leading cause of decrease in life expectancy due to disability and the second cause of death worldwide [1,2,3]. These processes are growing at a higher rate than the rest of the human diseases due to the aging of the population. The distribution of years of life lost due to disability from neurological diseases is age-dependent, increasing notably after 50 years [1]. Contrary to the opinion that the prevalence of neurological diseases is more characteristic of Western and more developed countries, when the years of life lost due to disability are adjusted for age, it can be verified that the distribution is similar across the world, regardless of the level of development [4].

Despite the increase in knowledge and the implementation of more and better health care, mortality from neurological diseases increased by 39% and years of life lost due to disability by 15% between 1990 and 2016 [1]. The introduction of preventive practices, especially the control of arterial hypertension, the change in social attitude towards health care and the inclusion of more effective treatments, have achieved a decrease in mortality and disability due to stroke [5,6]. However, the aging of the population [7] will cause an increase in the incidence of stroke in the coming decades [6,8]. To these dramatic incidences of stroke, we must add 10 million patients in Europe who have developed dementia, of which at least half are of vascular origin [8]. The socioeconomic repercussions of stroke are very significant. In 2010, the cost of strokes in Europe was estimated at EUR 905 billion, 60% of which were direct costs. The human cost for individuals, as well as the social cost for their families and communities, is incalculable.

It is well established that the only pharmacological treatment with proven efficacy in the acute phase of ischemic stroke is reperfusion through systemic fibrinolysis, mechanical thrombectomy, or a combination of both. Although mechanical thrombectomy is the preferred treatment [9], its usefulness is limited because of its application to proximal occlusions of the large arteries (about 10% of patients with ischemic stroke) and the need for centers with specialised staff and technology. On the other hand, the comparison of mechanical thrombectomy alone or associated with systemic fibrinolysis has not shown superiority [10,11]. In addition, intravenous or mechanical reperfusion treatment causes a benefit, assessed as an independent functional status at 3 months, of approximately 50% in patients who receive it, which depends on the type of stroke, the time since the onset of symptoms and the quality of the health facilities, which tends to be associated with the level of development of the country. In the best scenario, the patient is permanently left with some disabling injury. For intracerebral haemorrhage, there is no available pharmacological treatment with demonstrated efficacy [12].

In recent decades, attempts have been made to identify modifiable risk factors in the primary prevention of stroke. There is evidence–largely inconclusive–that has guided the management of patients at risk of developing acute episodes of cerebrovascular disease: encouragement of a healthier lifestyle; control of arterial hypertension, hyperlipidaemia, and diabetes; antiplatelet and anticoagulant treatments; and asymptomatic carotid and vertebral stenosis. However, despite extensive research, little solid evidence exists. The arterial hypertension reduction [13,14] and the new anticoagulants in selected groups of patients with atrial fibrillation [15] have shown clear clinical improvements. The correction of other metabolic risk factors does not provide the same level of evidence [16,17,18,19]. Indeed, the efficacy of arterial hypertension control is limited by low compliance with antihypertensive treatment, which is only a quarter in patients in high-income countries and three-quarters in low- and middle-income countries [20,21]. Another problem related to antihypertensive treatment is the possible association with worsening cognitive impairment in patients with brain aging [22,23].

Taking into account that stroke (ischemic stroke and haemorrhagic stroke) is a major health-related challenge, and its incidence will continue to rise in the coming years, early individual identification and treatment would: (i) save lives; (ii) reduce disability and improve quality of life; (iii) promote new therapeutic strategies; and (iv) reduce the economic burden of health care services. For all these reasons, it is necessary to search for new, more effective, and global paradigms to prevent cerebrovascular disease. The blood-brain barrier (BBB) breakdown with structural and functional changes in brain regions during acute ischemic stroke is critical. It remains unclear, however, whether BBB dysfunction is one of the first events that leads to brain disease or a down-stream consequence. In this review, our focus will be therefore on the BBB dysfunction associated with cerebrovascular disease and the added difficulty of its association with the deleterious or reparative effect, depending on whether the stroke is at an earlier or later phase. Once the structure and function of the BBB is outlined, we will look at the spatiotemporal chronic, slow, and progressive alteration of the BBB related to ischemic stroke. Finally, we propose a new perspective on preventive therapeutic strategies associated with brain aging based on targeting specific components of the BBB.

## 2. BBB Structure and Function

The BBB is a partially dynamic anatomical-functional structure that allows blood to be separated from the brain differentially in various parts of the brain parenchyma and in its communication with the cerebrospinal fluid (CBF) [24]. The bloodstream carries oxygen, nutrients, growth factors, and hormones that coordinate the functioning of the entire body, as well as the complex immune defense system. At the same time, the bloodstream also removes carbon dioxide and metabolic “waste” products from the central nervous system (CNS) [25]. The vascular tree is made up of arteries, arterioles, and the capillary bed, which is responsible for tissue exchange, and subsequently of venules and veins that allow tissue drainage. The microvascularization provided by the capillaries and the postcapillary venules has exclusive peculiarities in the CNS. The capillary network can be made up of non-fenestrated capillaries with a continuous basement membrane, or capillaries and continuous basement membrane, but fenestrated, or discontinuous capillaries that open gaps between the tissue and blood components [25,26].

The BBB is a unique structure in the entire body, exclusive to the CNS. It is made up of continuous and non-fenestrated capillaries, but which also have additional characteristics that further restrict the exchange between the brain and the vascular space [27,28,29]. The BBB protects the brain from compounds and molecules in the bloodstream by allowing only oxygen, glucose, amino acids, and other essential nutrients to cross it. The transport in the BBB can be due to selective transport or by passive diffusion that depends on adenosine triphosphate (ATP) and other metabolized elements from the blood towards the nervous tissue and vice versa [30]. The BBB does not include the entire CNS, and it has different peculiarities in its relationship with the CBF. It is not found in several areas of the CNS such as in some circumventricular organs, the roof of the third and fourth ventricles, the roof of the dicephalus, and the pineal gland [31,32,33,34].

### 2.1. BBB Molecular Components: The Neurovascular Unit

**Figure 1:**
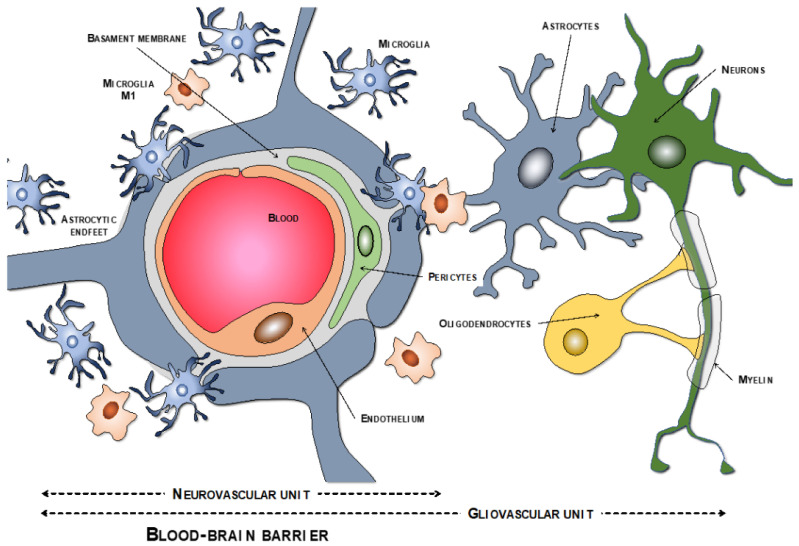
Components of the neurovascular and the gliovascular units of the BBB.

**Figure 2:**
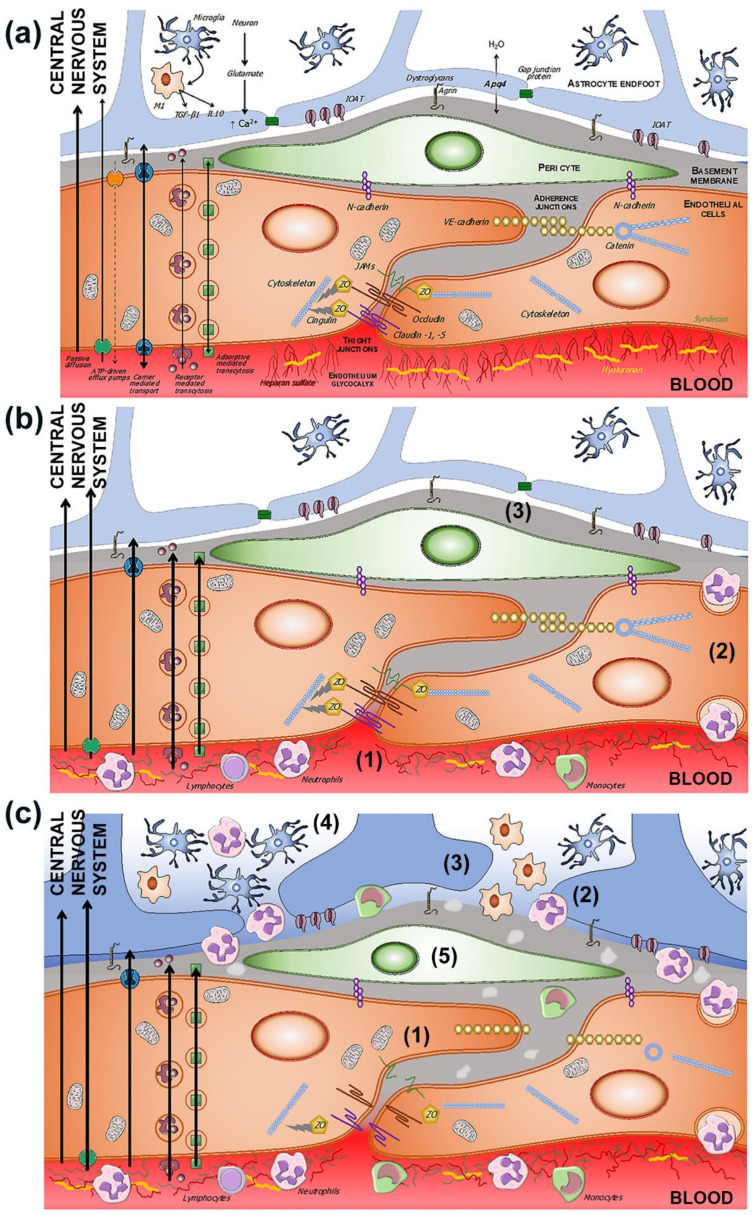
(**a**) Components of the BBB and the neurovascular unit. The endothelium glycocalyx protects the vascular endothelium and prevents the adhesion of blood cells. The endothelium is impermeable through the paracellular spaces by means of a complex system of proteins that form the tight junctions (TJ). The adherent junctions facilitate the flexibility of the capillary to changes in the diameter of the vascular lumen. Through the endothelium, the passage of molecules necessary for the functioning of the CNS is allowed by various mechanisms, some passive and others linked to exchange or receptor pumps. Pericytes embedded in the basement membrane, astrocytic endfeet, and microglia complete the BBB. (**b**) BBB dysfunction in situations of chronic and subclinical cerebral ischemia. The increase in permeability is due primarily to the breakdown of the endothelium glycocalyx (1), which facilitates the adhesion and penetration of leukocytes through the endothelium (2), and the alteration of the selective transcellular permeability system, which allows the entry of water and proteins into the perivascular space (3). (**c**) Hours after the onset of acute cerebral ischemia, tight and adherens junctions break down (1), leukocyte invasion (2), astrocytic endfeet oedema (3), microglia proliferation and activation (4), and pericyte dysfunction (5).

The endothelial cells are described as the main cells involved in the structure of the BBB. However, these cells are not by themselves capable of carrying out all the functions that the BBB must perform. Thus, close anatomical and functional contact with the pericytes is required. Both types of cells are surrounded by the basement membrane, constituting a vascular unit (Figure 1). Outside it, the vascular basement membrane is reinforced with a parenchymal matrix where the feet of astrocytes rest, which are accompanied by cells with immune functions, such as microglia and macrophages, and neurons. All this cellular functional group constitutes the neurovascular unit (Figure 2a). Therefore, the normal functioning of the BBB requires an adequate interaction between a luminal component, consisting of specific endothelial cells reinforced by pericytes and a vascular basement membrane, and an abluminal component consisting of an extracellular matrix, astrocytes, microglia, macrophages, and neurons [34,35,36,37].

#### 2.1.1. Endothelial Cells

A continuous, non-fenestrated, strongly attached differentiated endothelium lining the entire luminal portion of the capillary constitutes the fundamental element of the BBB. This endothelium is specialised to restrict the paracellular and transcellular movements of solutes [34]. Functionally, the endothelial cells of the capillaries that nourish the CNS are intimately linked to each other by tight junctions (TJ). They have minimal pinocytotic activity, without expression of cell adhesion proteins, and a large concentration of mitochondria that generate enough energy for the activity of membrane receptor channels that will transport metabolically active products, such as glucose, amino acids, ions, nucleosides, nucleotides, and monocarboxylic substances [24,36].

The luminal surface of the endothelium is covered by a fragile extracellular structure endowed with great functional dynamism, called the endothelial glycocalyx [38]. This area acts as an interface between the lumen of the vessel and the endothelium. It also provides it with plasticity and adaptability to the environment and constitutes the BBB’s first line of defense [39]. The endothelial glycocalyx is like a brush with a negative charge that protects the endothelium, repelling contact with potentially harmful molecules for the CNS as well as avoiding the adherence and approximation of circulating cells to the endothelial surface. This layer is made up of glycoproteins and proteoglycans (heparan sulfate, chondroitin sulfate, and hyaluronic acid) [40,41]. Both are proteins linked by covalent bonds to sugar chains. Two layers of endothelial glycocalyx are identified: one is more luminal and wider, consisting mainly of heparan sulfate; and another, deeper and denser, which is attached to the endothelial surface, of chondroitin sulfate and hyaluronic acid [38]. The endothelial glycocalyx is anchored to the endothelium through a family of transmembrane proteins called syndecans, which link glycoproteins and proteoglycans with proteins of the endothelial cytoskeleton, such as actinin, tubulin, and others [42].

#### 2.1.2. Tight Junction

The endothelium that constitutes the BBB is adhered through TJ, formed by a complex network of transmembrane proteins, which provides great resistance to the passage of molecules that can damage the CNS [43,44]. There are also adherent junctions that increase the stability and integrity of the endothelial layer and with the peri-endothelial cells that constitute the neurovascular unit [29]. TJ is made up of three families of membrane proteins: the claudins, occludin, and junctional adhesion molecules [45]. All this molecular framework is associated with cytoplasmic proteins, including the zonula occludens −1, −2, and −3 (ZO-1, ZO-2, and ZO-3), cingulin, and others. These cytoplasmic proteins bind to membrane proteins of the endothelial cytoskeleton, which favours the structural and functional integrity of the luminal surface of the capillary [46,47,48].

Claudins are phosphoproteins with four transmembrane domains, and claudin 1 and 5 constitute the main components of the TJ of the cerebrovascular endothelium. These claudins are bound to cytoplasmic proteins, including ZO-1, ZO-2, and ZO-3 [49]. Occludin is a larger protein than claudins, also with four extracellular domains and bound to cytoplasmic proteins. Claudins and occludin constitute the main molecular elements of the TJ of endothelial cells [25]. Junctional adhesion molecules belong to the immunoglobulin superfamily, with a single transcellular domain whose function is not well defined, but they also bind to actin to stabilize endothelial cell junctions [29]. Cadherins are the main components of adherent junctions. For this complex structural and functional scaffolding of the CNS capillary luminal layer, VE-cadherin interacts with ZO-1 and catenins [50].

#### 2.1.3. Pericytes

Pericytes are mural cells similar to smooth muscle cells that surround arterioles and arteries, but unlike them, their embryological origin is ectodermal and not mesodermal [51]. These cells, embedded in the basement membrane, form a discontinuous layer on the abluminal surface of the endothelium. Pericytes, although not adhered to the CNS endothelium, are attached through sporadic peg-and-socket junctions mediated by N-cadherins [52]. Adhesion plaques, gap junctions, and TJ, which facilitate the exchange of ions, metabolites, second messengers, and ribonucleic acids between the two types of cells [53], have also been demonstrated [25]. Pericytes participate in the stability and integrity of the neurovascular unit. It has been shown that they are important in the development of the BBB, in angiogenesis, and can even act as stem cells of the CNS [53,54]. Likewise, pericytes contain contractile proteins and express receptors for substances that participate in vascular tone, such as catecholamines, angiotensin, vasopressin, and endothelin-1 [29]. Through these multiple mechanisms, pericytes can control the diameter of capillaries and participate in the autoregulation of cerebral blood flow. In addition to the close interrelationship between endothelial cells and pericytes, signaling pathways between astrocytes and pericytes actively participate in the integrity and normal functioning of the BBB [28].

#### 2.1.4. Basement Membrane

The extracellular matrix components that constitute the basement membrane are essential for the stability and integrity of the BBB. This membrane completely surrounds the vascular tube, lines the abluminal face of the endothelium and separates it from the astrocytic feet. Pericytes are housed within the basement membrane. The basement membrane is made up of two superimposed layers, one internal vascular and the other external parenchymal [25]. The vascular extracellular matrix is secreted by the endothelium and pericytes, while the parenchymal matrix is produced by astrocytes. Although both layers are made up of collagen, fibronectin, proteoglycans, glycoproteins, and laminins, their composition is not identical [55,56]. The basement membrane is not only an element that gives consistency to the neurovascular unit and prevents the passage of many molecules, but also actively participates in the repair of the BBB [57,58]. Receptors are anchored in the basement membrane, mainly dystroglycans and various members of the integrin family of adhesion receptors [44]. It is also an important source for various growth factors [44].

#### 2.1.5. Astrocytes

Astrocytes constitute the most abundant cell type in the CNS of mammals, and their extensions towards neurons and capillaries allow the establishment of a close relationship between neuronal activity and cerebral perfusion [59]. Astrocytic endfeet completely surround the surface of the cerebral capillaries, resting on the parenchymal layer of the basement membrane. At the junction point of these two structures there is a high density of intramembranous organic anion transporters essential for the proper functioning of the BBB [60,61]. The astrocytic endfeet express proteins, such as dystroglycans, that anchor them to the basement membrane through agrin, which stabilizes this junction and the neurovascular unit [60]. Other proteins expressed by astrocytes are aquaporin-4 and potassium channels, which regulate water homeostasis in the CNS [62,63,64]. Aquaporins also participate in the glymphatic system that facilitates the flow of interstitial fluids through the BBB [65,66,67]. Proteins, such as sonic hedgehog, retinoic acid, and angiopoietin-1, contribute to the stability, functionality, and repair of the BBB and the differentiation of astrocytes [44].

The astrocytes of the neurovascular unit are essential in the relationship between neuronal activity and cerebral blood flow, regulating the contraction and relaxation of the pericytes in the neurovascular unit and of the smooth muscles in the arterioles of the CNS [59,64]. Other functions associated with the normal functioning of the astrocytic endfeet are the regulation of pH and its participation in the reuptake of neurotransmitters and in the control of excitotoxicity by glutamate [68,69,70,71,72]. The regulation of neurotransmitter concentration is an essential function of the astrocytic endfeet and the BBB. Under normal conditions, the BBB is impermeable to the passage of amino acid neurotransmitters from the blood to the CNS; however, the release of these excitatory neurotransmitters into the bloodstream is regulated by Na+-dependent and -independent transporters that actively control excitotoxicity levels in both normal and pathological situations [73].

#### 2.1.6. Macrophages and Microglia

The CNS is a privileged space from an immunological point of view, and in normal situations, the BBB is impermeable to the passage of immune cells. In the embryological phase, some cells with immunological capacity are located in the perivascular space of the CNS. The macrophages responsible for the first line of defense in cases of altered BBB permeability are located in arterioles located in the Virchow-Robin space, between the astrocytic endfeet and the vascular wall [74].

Microglia are immune cells that constitute up to 10–15% of glial cells. They are preferably located in the perivascular space of the CNS capillaries, and because of their importance in the integrity and regulation of the BBB, they form part of the neurovascular unit [75]. In normal situations, microglia are in a state of rest, immobile, without endocytic or phagocytotic activity, although with multiple cytoplasmic extensions in the extracellular medium of the CNS, which allow pathological or toxic mediators to be detected [76]. In these non-physiological situations, microglia are activated in two very different ways: M1 and M2. M1 microglia have pro-inflammatory functions and facilitate increased BBB permeability, while M2 microglia are immunoregulatory, anti-inflammatory, and reparative of the BBB. It also has the phagocytic capacity to remove cellular debris and contribute to the repair of neurons [76,77].

The mechanisms that can condition the two types of microglial activation are not well known, although this activation may be related to the severity of pathological aggression [78]. Astrocytic endfeet and pericytes exchange multiple mediators that can activate or modify microglia differentiation. Similarly, microglia are capable of influencing other cellular components of the neurovascular unit [79,80]. Microglia release interleukin-10 (IL-10) and transform growth factor-beta 1 (TGF-ß1) in the same way as astrocytic endfeet [76]. The endothelium of the BBB possesses abundant receptors for TGF-ß1, which play a critical role in the integrity of the BBB [81].

### 2.2. Transport Pathways across the BBB

The nutritional and metabolic needs of the CNS require that the BBB play a containment role against potentially harmful substances, but at the same time, it should be permeable to other necessary substances. In an intact BBB, this permeability occurs through four physiological mechanisms: passive diffusion, active efflux, transporter-mediated mobility, and receptor-mediated transport. In situations in which the BBB is affected, other mechanisms increase the exchange of substances from the blood to the CNS and vice versa [29].

#### 2.2.1. Passive Diffusion

Small lipid molecules can diffuse across the endothelium and into the CNS. The facility for this transfer depends on lipid solubility, a size of less than 400 Da and with less than six hydrogen bonds. However, a limited number of drugs that meet these requirements fail to cross the BBB, which suggests that other, yet unknown mechanisms are involved [29].

#### 2.2.2. Active Efflux

Mobility of some molecules across the endothelium can also be achieved using ATP-driven efflux pumps located in the luminal layer of the capillary. The most representative molecules are the P-glycoproteins, the multidrug resistance proteins, and the breast cancer resistance proteins. Some isoforms of the multidrug resistance proteins can also be located in the abluminal membrane of the endothelium, which explains the bidirectional flow that some molecules can have through the cerebrovascular endothelium [82,83].

#### 2.2.3. Carrier-Mediated Transport

The BBB cannot prevent the passage of essential substances for the normal functioning of the CNS, and this is partly achieved through other routes such as solute transporters. More than 300 transporter genes are known to encode proteins that are expressed in the endothelial membranes, which facilitate the transport of a wide variety of molecules, such as amino acids, fatty acids, monocarboxylic acids, hormones, nucleotides, choline, and vitamins [82,84,85]. Some of these transporters are expressed in both parts of the endothelial membrane, and others only in the luminal or abluminal membrane, which in some cases conditions the preferential transport of these substances to the CNS or to the blood [24,86]. TJ, through its ability to facilitate the passage of lipid rafts, preserves the essential polarity of the BBB and corrects its possible alteration caused by the operation of carrier-mediated transport in one direction or another [87].

#### 2.2.4. Receptor-Mediated Transport

The BBB is impermeable to the passage of large peptides and proteins. However, the CNS requires some neuropeptides, hormones, and growth factors for its normal functioning [86]. This is achieved through mechanisms of transcytosis, which is a form of endocytosis [25,26,27,29]. Although in the CNS transcytosis is much more limited than in the rest of the capillaries, the process works for large molecules. The mechanism of integration of these molecules in the endothelium is achieved through the formation of caveolae or vesicles from the luminal and/or abluminal membranes of the endothelium, which cross the endothelial cell and are transported to the opposite side [88]. There are two types of transcytotic mechanisms of caveolar transport: receptor-mediated transcytosis and adsorptive-mediated transcytosis.

In the first case, the macromolecules bind to specific receptors located on the cell surface. Vesicles are formed that are internalized in the endothelial cell and are exocytosed on the opposite cell surface. This mechanism is used by insulin, transferrin, some immunoglobulins, and low-density lipoproteins [89,90]. A special form of receptor is constituted by the major facilitator superfamily that allows the passage of omega-3 fatty acids, essential for the CNS. Likewise, this transport has the mission of maintaining the integrity of the BBB [91,92]. In the other mechanism of transcytosis, molecules with a strong electrical charge interact with specific areas of the cell membrane and are included in the caveolae to traverse the thickness of the cell [87]. The number of vesicles, or caveolae, of the CNS endothelium is much lower than that of endothelia in other parts of the body. At the same time, these vesicles must avoid the lysosomal compartment for successful transcytosis. This ability for vesicles to avoid lysosomes appears to be another mechanism unique to the BBB endothelium [88,93].

## 3. Evolution of BBB Dysfunction in the Setting of Ischemic Stroke

The terms “BBB disruption or BBB breakdown” suggest the destruction of an anatomical barrier resulting in the disappearance of the separation between the vascular component and the CNS parenchyma. However, the BBB is a complex functional system in which cellular elements and extracellular matrices participate with multiple mechanisms that allow both components to become independent and interrelated, and its disruption does not necessarily mean that its constituting anatomical elements have disappeared or been destroyed. As a result, the term "BBB dysfunction" appears more appropriate to describe the changes that occur when any of the mechanisms is altered in a process that can be abrupt at times but can also be dynamic and progressive (Table 1).

In rare monogenetic cerebrovascular diseases, the specific alteration of some structural element or some specific function of the BBB may constitute the central element, as in cerebral autosomal dominant arteriopathy with subcortical infarcts and leukoencephalopathy (CADASIL), Allan-Herndon-Dudley syndrome, Alexander disease, Nasu–Hakkola disease, familial cavernomas, Fahr’s disease, lysosomal storage diseases, and others [28]. However, BBB dysfunction is the cause or consequence of much more frequent neurological processes, such as Alzheimer’s disease, Parkinson’s disease, amyotrophic lateral sclerosis, dementia associated with human deficiency virus infection (HIV-1), head injuries, ischemic and haemorrhagic stroke, epilepsy, brain tumours, multiple sclerosis, encephalopathies, and encephalitis [24]. An unresolved question is whether BBB dysfunction is the cause, the consequence, or both of many neurological diseases.

In cerebral ischemia, BBB dysfunction can be both the cause and the consequence of increased permeability, as well as the greater or lesser degree of vasogenic oedema or haemorrhagic transformation, associated or not with reperfusion treatments, which characterises the cerebrovascular disease, depending both on the intensity of the ischemia and/or hypoxia, and on the speed of its onset [25,109].

The involvement of the various components of the neurovascular unit is not the same in all ischemic situations. “Chronic” ischemia mainly affects the capillaries of the territories of the penetrating arteries, located in the white matter. In contrast, acute ischemia is the result of the occlusion of much larger caliber arteries. These differences may explain the different mechanisms of BBB dysfunction [86,88,109,110,111,112,113]. The increased permeability of the BBB associated with cerebral ischemia has implicated the structuring of tight and adherent junctions, which would facilitate increased transport of cells, molecules, and solutes through these endothelial intercellular spaces [94,109,111,114,115,116] (Figure 2b). However, other studies have failed to show the alteration of the TJ, either in chronic hypoxia or in the initial phases of acute cerebral ischemia [87,88].

### 3.1. Endothelials Cells

The alteration of the electrostatic barrier formed by the glycocalyx may be the first event that initiates a cascade of processes that ends in a complete dysfunction of the BBB [116] (Figure 2b). High blood pressure and diabetes can alter the properties of the glycocalyx. Several works suggest that this early alteration of the glycocalyx prevents the activity of endothelial nitric oxide synthase (eNOS), which would induce a decrease in capillary flow and facilitate shear stress [94]. These alterations increase the adherence of blood cells to the endothelial surface [95] and allow the release of adhesion molecules such as vascular cell adhesion molecules (VCAMs), intracellular adhesion molecules (ICAMs), and platelet-endothelial cell adhesion molecules (PECAMs) [96]. Therefore, the degradation of glycocalyx initiates increased permeability and dysfunction of the BBB, allowing contact of blood cells with the endothelium, loss of vascular reactivity, and cerebral oedema, and fails to protect the endothelium from oxidative stress, both of local and systemic origin [95,97] (Figure 2b).

### 3.2. Microglia

The interaction between the leukocytes and the endothelium contributes to the increase in the alteration of the glycocalyx. In addition, the adhesion of neutrophils to the endothelium is the primary cause of increased BBB permeability, first transcellular and then intercellular [98,99]. BBB dysfunction is enhanced by neutrophils through the production of reactive oxygen species, proteases, and neutrophil extracellular traps [100]. It should be noted that monocytes also contribute to endothelial dysfunction in the first phase [99,101]. Furthermore, proteases released by neutrophils and other leukocyte cells facilitate their transcellular diapedesis and thus reach the CNS [113], where they are initially phagocytosed by microglia [76] and subsequently invade the perivascular space.

### 3.3. Basement Membrane

In these early phases, caveolar transcellular transport activity is increased, both through receptor-mediated and adsorptive-mediated transcytosis [88,117]. A few hours after the onset of cerebral ischemia, a rapid increase in endothelial caveolae is confirmed [102], with no evidence of structural or functional alterations in the tight and adherent junctions [118]. Endothelial transcytosis causes an increase in water content in the perivascular area of the neurovascular unit, already in very early stages of cerebral ischemia, even in subclinical forms [118,119]. The formation of endothelial caveolae responsible for increased transcytosis and endothelial permeability has been associated with increased expression of caveolin, a constitutive protein of vesicles [88]. These caveolae are not only expressed in the endothelium, but also in the pericytes and in the basement membrane and have been related to astrocytic endfeet oedema [102,107,108] (Figure 2b,c).

Although in subclinical cerebral ischemia it is possible to hypothesise that BBB dysfunction is limited to an increase in intracellular endothelial transcytosis [110,120], a biphasic response has clearly been demonstrated in strokes: an initial phase (a few hours after the onset of the decrease in cerebral blood flow below the threshold that allows the survival of neurons) characterised by the transcellular alterations that we have just described, followed by a later phase (two or more days after the onset of the stroke) of intercellular predominance, characterised by the destructuring of the constitutive proteins of the TJ and of the adherent junctions. These alterations are more destructive, responsible for the greater proportion of vasogenic oedema, haemorrhagic transformation, and, occasionally, malignant cerebral oedema associated with extensive cerebral infarctions [34,87,111,115,117,118,121,122].

### 3.4. Tight Junction

Disruption of tight and adherent junctions is mainly triggered by metalloproteases activated by mechanisms associated with hypoxia-inducible factor-1α and by cytokines [87,123,124]. Oxidative stress is another factor that facilitates the increase in intercellular permeability, and therefore TJ are more affected in situations of ischemia-reperfusion [125] (Figure 2c). The alteration of the TJ ends up being the result of the decrease in the expression and the change in location of the families of claudins, occludins, junctional adhesion molecules, and cadherins [103,104]. A decrease in the expression of cytoplasmic proteins, such as the ZO and of the cytoskeleton that anchor the proteins of the junctions to the cerebral endothelium, has also been demonstrated [105,106] (Figure 2c).

BBB dysfunction is common in all forms of cerebral ischemia and many other neurological diseases. However, the intensity and time of the appearance of this dysfunction vary. In subclinical cerebral ischemia, the dysfunction of the BBB is fundamentally transcellular, with the passage of molecules and cells in certain territories, fundamentally of the white matter [126]. In these situations, the perivascular inflammation is discreet, and although there is an increase in perivascular water, there is no space problem, and the oedema is limited.

### 3.5. BBB Dysfunction and Transport Mechanisms

BBB dysfunction is one of the hallmarks of ischemic stroke pathology and is characterised by changes in TJ protein complexes (leading to increased paracellular solute leak), modulation of transport proteins and endocytotic transport mechanisms (changing transcellular transport for some substances), and inflammatory damage. Such barrier failure results in a notable increase in paracellular permeability at the level of the cerebral microvasculature [87,127]. In an ischemic stroke, in the early stages, and as a consequence of the release of glutamate [128,129,130,131], sodium and water enter the neurons, and a very early cytotoxic oedema occurs, although the BBB is still intact. In the following hours and days, and as a consequence of the intense transcellular and intercellular dysfunction of the BBB, an intense vascular oedema originates, which affects both the grey and white matter, responsible for the increase in morbidity and mortality due to stroke. This vasogenic oedema is largely the result of inflammatory mechanisms.

### 3.6. Neuroinflammation in BBB Dysfunction

All constitutive cell types of the neurovascular unit are affected by BBB dysfunction; its implications depend on the intensity and time of evolution of the triggering cause. Neuroinflammation affects all cellular elements of the neurovascular unit, but microglia and astrocytes have the ability to express multiple mediators of inflammation, in a greater proportion than the endothelium or pericytes. The result of neuroinflammation facilitates leukocyte adhesion, transmission, and infiltration. This inflammatory process will be responsible, in the first phase, for the vasogenic oedema and the greater degree of involvement of the BBB dysfunction, but later it will contribute to the repair of the BBB and to the healing of the brain injury [132,133].

#### 3.6.1. Cytokines

Cytokines are small pleiotropic polypeptides capable of regulating innate and acquired immune responses [134,135]. Among them, interleukin 1-β (IL-1β), tumour necrosis factor-α (TNF-α) and interleukin-6 (IL-6) are expressed in the first hours after severe cerebral ischemia and participate in the increased permeability of the BBB. Multiple studies confirm this implication, both in experimental models and in human clinical practice [136,137,138,139,140,141,142,143,144]. However, interleukin-10 (IL-10), interferon-β (IFN-β), and transforming growth factor-β (TGF-β) can modulate to inhibit the inflammatory response, decrease BBB dysfunction, and facilitate recovery from injury [145,146,147].

Among the cytokines, the tumour necrosis factor-like weak inducer of apoptosis (TWEAK), belonging to the TNF superfamily, has shown its participation in the immune response and inflammation through its union with its specific receptor, fibroblast growth factor-inducible 14 (Fn14), which is a type I transmembrane protein belonging to the superfamily of TNF receptors. The TWEAK exists in two forms: the mTWEAK, which is a type II transmembrane protein, consisting of 249 amino acids; and a soluble form, the 156 amino acid sTWEAK, resulting from the proteolysis of the complete transmembrane protein [148,149,150,151,152].

The TWEAK/Fn14 complex needs to trimerize to activate multiple molecular pathways. It is especially dependent on the canonical and non-canonical nuclear factor-kappa B (NF-κB) pathways, but also on the mitogen-activated protein kinase (MAPK), extracellular signal-related kinase (ERK), activator protein-1 (AP-1), and p38 cascade pathways [153]. The TWEAK/Fn14 mechanism has been shown to be related to several biological, pro-inflammatory, and even reparative responses depending on the cell type (endothelium, pericytes, and microglia) and on their concentration [154]. The expression of Fn14 is low in normal situations, but increases rapidly after exposure to various stimuli, such as ischemia, oxidative stress, or inflammation [155]. The response may also vary in relation to the different expression of Fn14 (possibly elevated in situations of chronic ischemia) and of TWEAK (possibly low during chronic ischemia but rapidly upregulated in acute ischemia). The increased expression and trimerization of TWEAK/Fn14 increases the production of metalloproteinases (MMPs) and alters the permeability of the BBB [150,152,153,154,155,156]. MMPs destroy collagen, laminin, and fibronectin, proteins that make up the basal membrane of the neurovascular unit [134], but also proteins of the TJ of the endothelium.

Recently, we have confirmed the involvement of sTWEAK expression in patients with subclinical cerebral micropangiopathy [157], in the recurrence of ischemic stroke [158], in the haemorrhagic transformation of patients undergoing reperfusion treatments [159], and in the growth of intracerebral hemorrhages [160].

Chemokines are low molecular weight proteins that are primarily responsible for recruitment, adhesion, and infiltration of leukocytes through the BBB to the perivascular space and brain parenchyma [134,161]. Chemokines are expressed primarily by astrocytes and microglia in response to elevated proinflammatory cytokines [162]. The most expressed chemokines in relation to cerebral ischemia are monocyte chemoattractant protein-1 (MCP-1) and stromal cell-derived factor-1 (SDF-1) [163,164]. Despite the clear involvement of these molecules in the neuroinflammatory response associated with BBB dysfunction and vasogenic oedema, they are also capable of activating and attracting bone marrow stromal cells to the infarcted area, thus facilitating the healing of stroke [165].

Selectins, the superfamily of immunoglobulins (ICAM, VCAM, and PECAM) and integrins, are a group of inflammatory mediators responsible for the infiltration of neutrophils into the brain parenchyma and are essential for these cells to cross the cerebrovascular endothelium through a process of transcellular diapedesis or through the intercellular spaces altered by the destruction of the TJ [133,166].

#### 3.6.2. Ischemic Neurons

In general, the focus is on the endothelium of the cerebral microvascularization, the origin and the end of the processes that condition the dysfunction of the BBB. However, ischemic neurons also play an important role and condition processes that contribute to the functional and structural alteration of the BBB.

Oxidative stress caused by reactive oxygen species participates in ischemic brain injury, contributes to the destruction of neuronal membranes, and accelerates BBB dysfunction [134,167,168,169]. One of the sources responsible for superoxide radicals are leukocytes, whose proliferation, adhesion, and penetration in the neurovascular unit and in the brain parenchyma had already started by then. The other source of reactive free radicals is produced by some isoforms of nitric oxide synthases (NOS). With the exception of the endothelial isoform (eNOS), which exerts a vasodilator and protective effect, the other two isoforms, the neuronal one (nNOS) and, especially, the inducible one (iNOS), cause high levels of nitric oxide that reacts with excess superoxide to produce peroxynitrite. These toxic isoforms are expressed by extravasated neutrophils, by microglia, and also by neurons affected by acute cerebral ischemia [170,171].

Although vascular endothelial growth factor (VEGF) has proangiogenic activity, in situations of acute cerebral ischemia, affected neurons induce VEGF expression in astrocytes and are responsible for the involvement of tight and adherens junction proteins [109,172,173].

Many mechanisms involved in BBB dysfunction, especially neuroinflammatory mechanisms, give rise to increased expression of MMPs, which have been shown to be involved in the destruction of the basement membrane of the neurovascular unit, both in experimental models [174,175,176,177], as in the human clinic [178,179,180,181].

Other molecules with structural and signalling functions called ceramides have been described to be involved in stroke [182]. In this regard, high levels of long-chain ceramide have been associated with BBB disruption and disfunction [183,184]. Furthermore, elevated levels of ceramide were recently described in plasma samples from stroke patients with large artery atherosclerosis and cerebral small vessel disease [185].

### 3.7. Neuroimaging of BBB Dysfunction

**Figure 3:**
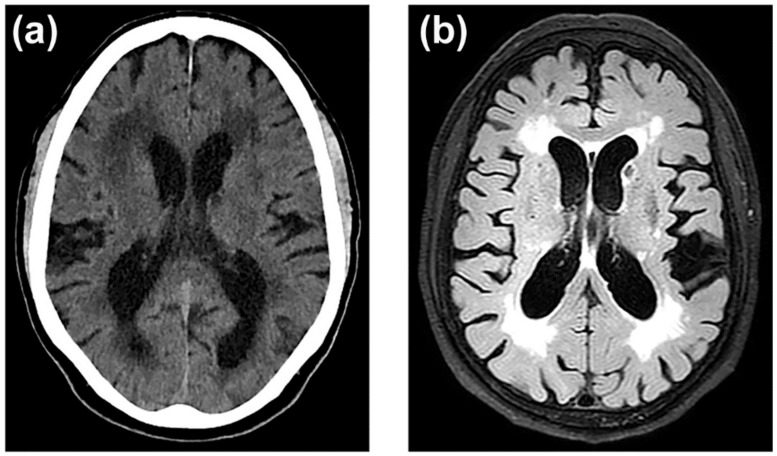
Leukoaraiosis in a 67-year-old hypertensive patient with occasional headaches. (**a**) CT showing diffuse hypodensity (rarefaction) of the periventricular white matter. (**b**) T2-MRI of the same patient, with sharper hypersignals.

In 1987, Hachinski et al. [186] coined the term leukoaraiosis to define a rarefaction of the periventricular white matter that appeared as irregular and diffuse areas of hypodensity on computed tomography (CT) images. With the introduction of magnetic resonance (MRI), these images became more apparent with hypersignals on T2-weighted or FLAIR MRI [187] (Figure 3).

Leukoaraiosis has been related to chronic and subclinical cerebral hypoxia-ischemia associated with BBB dysfunction [126,188,189,190,191,192]. The clinical importance of this neuroimaging marker is that its prevalence affects 70% of adults over 65 years of age, especially women [191], and that its presence multiplies the risk of stroke by three, the risk of dementia by two [189], and the risk of intraventricular haemorrhage by one and thirty-eight hundredths [193]. We have also verified that the volume of leukoaraiosis has a direct relationship with the risk of developing clinical manifestations (from 1.7 times more frequent with grade I leukoaraiosis on the Fazecas scale [194], up to 4.7 times in those with grade III) [157]. The intensity of BBB dysfunction is also related to the development and volume of leukoaraiosis [190,195]. Although leukoaraiosis begins in the neurovascular unit with BBB dysfunction, its presence progressively involves oligodendrocyte precursors and oligodendrocytes that surround axons. This neurovascular unit extended to oligodendrocytes and myelinated axons is called the gliovascular unit, which constitutes the anatomical and functional substrate of leukoaraiosis [188,195]. The image of hypodensity in the CT or hypersignal in the MRI, characteristic of leukoaraiosis, implies the alteration of all the elements of the neurovascular unit, perivascular oedema, atrophy of the oligodendrocytes, rarefaction of myelin, degeneration of the axons, and reactive gliosis [196].

In an acute stroke, the neuroimaging of the first hours does not correspond to any dysfunction of the BBB. The interruption of cerebral blood flow below 20% of its normal values causes the failure of the sodium and potassium pumps, which leads to an influx of sodium and water into the cells, to the loss of the ionic gradient and to the depolarization of cell membranes. This process affects all cell types, but especially astrocytes. In addition to the failure of the ion exchange pumps, there is an increase in the release of neuronal glutamate, which activates the N-methyl-D-aspartate (NMDAR) and α-amino-3-hydroxy-5-methyl-4-isoxazolepropionic acid (AMPAR) receptors, and subsequently, the entry of calcium and sodium ions into postsynaptic neurons [197,198,199]; in addition to glutamate transporters, mainly type 2 (EAAT2), which, together with aquaporin-4, contribute to the glutamate uptake into astrocytes. Glutamate, through metabotropic receptors, also contributes to increased BBB permeability and astrocytic swelling [131,200]. This cytotoxic oedema, which is caused by the entry of water into the cells, does so at the expense of a decrease in extracellular water, so there is no increase in the net content of water in the brain, nor, therefore, does it cause an increase in the volume of the lesion [201,202]. Cytotoxic oedema produces a characteristic increased signal on diffusion-weighted MRI (DWI-MRI) [203].

**Figure 4:**
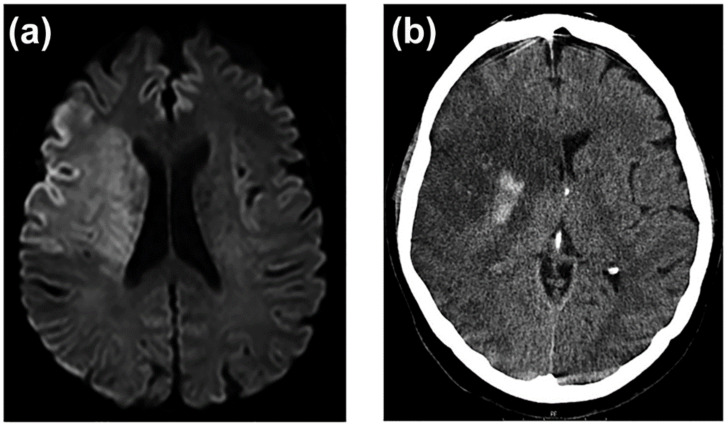
A 61-year-old woman with cardioembolic stroke in the territory of the middle cerebral artery. (**a**) In the first DWI-MRI (left) that corresponds to a cytotoxic oedema. (**b**) The same patient at 48 h (right). CT shows intense vasogenic oedema and hemorrhagic transformation of the infarct.

**Figure 5:**
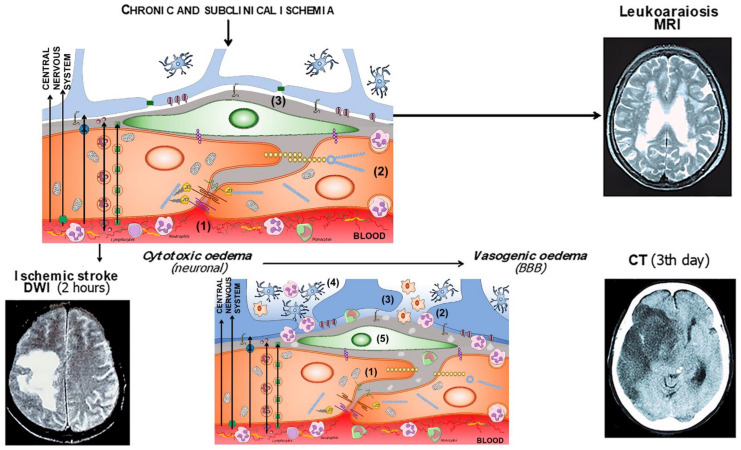
Correspondence of BBB alteration with neuroimaging in situations of chronic cerebral ischemia and the evolution of cytotoxic oedema to vasogenic oedema in acute cerebral ischemia.

After the first few hours of stroke, all the mechanisms that cause the dysfunction, and later the degradation, of the BBB [204] are progressively set in motion, with the consequent passage of water and proteins from the vascular space to the interstitial space, which causes vasogenic oedema. In contrast to cytotoxic oedema, vasogenic oedema is isosmotic and accumulates primarily in the extracellular compartment, causing a considerable increase in lesion volume. Reperfusion of ischemic tissue can increase the volume of vasogenic oedema [205] (Figure 4). The entry of water into the extracellular space causes the DWI-MRI signal to disappear, and it becomes more evident on CT and MRI on T2-weighted sequences [206]. Unlike neuroimaging of cytotoxic oedema, CT, or T2-weighted MRI images of vasogenic oedema are excellent markers of severe BBB disturbance (Figure 5).

## 4. BBB as a Target for Brain Aging and Cerebrovascular Disease Prevention

Currently, and despite the great development in early diagnosis of brain aging and therapeutic management of ischemic stroke, the results are modest: high morbidity, decreased life expectancy, and a mortality that fails to decrease. The availability of molecular and neuroimaging markers of chronic and subclinical cerebral ischemia opens the possibility of early therapeutic interventions to prevent the appearance of symptoms, which are almost always disabling and progressive. As chronic subclinical ischemia markers are associated with BBB dysfunction, research has intensified to discover their mechanisms and possible therapeutic targets to block them. All the cellular elements of the neurovascular and gliovascular units can be modified. In experimental models, both in vitro and in vivo, it has been possible to restore the functionality of the BBB, but in no case has benefit been shown in clinical trials [190,198,207,208,209]. In this line, identifying the differences between animal models and human cerebral vasculature, with potential implications for the search for new biomarkers and potential therapies, should be considered [210].

Drugs have been tested on the endothelium to restore the glycocalyx, prevent leukocyte adhesion, inhibit increased intracellular transport and transcytosis, maintain TJ integrity, and stimulate reparative neoangiogenesis [99,211]. Neuroimmunomodulators have also been tested to prevent leukocyte adhesion, penetration, and activation [98,212]. The inflammatory response is complex and affects the entire neuro- and gliovascular junction and participates in BBB dysfunction, for which many therapeutic targets have been explored [213]. Other approaches such as blocking of aquaporins in astrocytes and metalloprotease inhibitors, have been tried [87,103,199]. The role of microglia in BBB dysfunction has been the focus of much preclinical research [76,79,80]. Experimental studies have also been developed to block microglia activation and prevent BBB dysfunction through antioxidants, toll-like receptors, recombinant annexins, pinocembrin, minocycline, microRNAs, and others [76].

All the available evidence of the implication of TWEAK/Fn14 in cerebral ischemia, especially in BBB dysfunction, makes it a hopeful therapeutic target for situations of chronic cerebral ischemia, and also in the prevention of vasogenic oedema in acute cerebral ischemia [156,157,158,214,215,216]. TWEAK/Fn14 increases the permeability of the BBB by several mechanisms. Through classical NF-κB-mediated induction, it facilitates the expression of MMP-9, stimulates the transcellular transport of neutrophils through the endothelium, and activates microglia and astrocytes for the production of proinflammatory cytokines and chemokines [151]. The inhibition of TWEAK/Fn14 activity as a therapeutic target is facilitated by its extracellular accessibility and by the existence of different pharmacological approaches, such as anti-TWEAK antibodies, Fn14-Fc, anti-Fn14 antibodies, and Fc-TWEAK, which should be tested in preclinical studies.

### Nanomedicine and Drug Delivery Systems

The rapid expansion of nanotechnology for biological and medical applications has opened a new era in the diagnosis and treatment of many diseases. The use of nanoparticles (NPs) for clinical use has allowed the encapsulation of many drugs, a reduction in therapeutic doses, a reduction in adverse effects, increased drug stability, bioavailability, and targeting/delivery efficacy in the desired region, such as the BBB. Approximately 98% of small molecular weight drugs and almost 100% of larger molecular weight peptides and proteins do not cross the BBB. Nevertheless, the past few years have seen notable developments in systemic and local biomaterial-based nanosystems and microsystems drug delivery. The main types of NP systems used for drug delivery are liposomes, nanospheres, nanocapsules, dendrimers, micelles, and biomimetic nanostructures [217].

These new versatile NPs can simultaneously carry imaging agents (MRI or PET), therapeutic drugs, and targeting ligands to induce a controlled drug delivery that can be activated by internal (pH, temperature, redox) or external stimuli (light, temperature, magnetic field, ultrasound). Bony et al. reported a new NP to monitor alterations in the TJ protein expression (claudin-1) that may cause BBB leakiness through non-invasive MR imaging as well as a targeted delivery vehicle to improve site-specific target engagement of delivered therapeutics in old mice [218]. As examples of internal and external stimuli, Yang et al. showed a dual-targeted therapeutic strategy in vitro and in vivo reported to enable pH-sensitive drug release and that allows cerebral ischemia targeting to improve stroke therapeutic efficacy [219]. On the other hand, Correa-Paz et al. designed sonosensitive sub-micrometric capsules loaded with rtPA with a size of approximately 600 nm, synthesized using the layer-by-layer (LbL) technique, and coated with gelatine for clot targeting. This study evaluated the rtPA release by ultrasound (US)-responsive SCs and therapeutic effects in healthy and thromboembolic stroke model mice [220]. A recent review summarized applications and nanotechnology tools for the study of stroke and the research of novel therapies [221].

## 5. Conclusions and Future Perspectives

BBB dysfunction is pivotal in pathological processes of the small vessels in the brain that may be involved in chronic and acute cerebral ischemia, cognitive impairment, dementia, or gait disturbance. The cerebral endothelium represents a biological and mechanical barrier between the cerebral and vascular compartments. Consequently, this endothelium is probably the link between risk factors and vascular lesion. Neuroprotection to prevent infarct events is still an important objective in stroke research. There are currently no sensitive, specific, or precise biomarkers for accurately assessing BBB dysfunction, leukoaraiosis, or stroke risk. Understanding BBB age evolutions will help with new drug development and selecting the best performance window times, which could have a direct impact on clinical translation and personalized medicine. The versatility of nanomedicine has been widely exploited in the field of stroke. Its main objectives have focused on: (i) the diagnosis in acute phase; (ii) the improvement of the efficacy of thrombolytic therapy and protective drugs, and (iii) helping in the development of recovery therapies. However, no efforts have been made on stroke prevention. The use of nanotechnology could facilitate access to the BBB [218,222,223] and open new and effective therapeutic targets to prevent the progression of chronic cerebral ischemia, the appearance of ischemic and haemorrhagic strokes, and cognitive deterioration associated with brain aging. To this end, further in vitro and in vivo studies should be developed in order to improve their applicability in clinical studies.

## Figures and Tables

**Table 1:** Summary table of blood-brain barrier components affected by ischemic stroke.

Blood-Brain Barrier Components	Change/Event	References
Glycocalyx	Degradation	[94,95,96,97]
Endothelial cells	Interaction with leukocytes Increase in caveolae	[98,99,100,101,102]
Tight junctions	Decrease the expression/ change location of proteins	[103,104,105,106]
Pericytes	Increase in caveolae Astrocytic endfeet oedema	[102,107,108]
Basement membrane		
Astrocytic endfeet		

## Data Availability

Not applicable.
